# Supplementary material for: Identification of photoperiod-induced specific miRNAs in the adrenal glands of Sunite sheep (Ovis aries)
Source: Front Vet Sci. 2022 Jul 22;9:888207. doi: 10.3389/fvets.2022.888207 (PMC9354845; doi:10.3389/fvets.2022.888207)
Supplement: Supplementary file 1 [file Data_Sheet_1.ZIP › Supplementary file/miRDeep2 file.zip!/miRDeep2 file/Know_Structure/LP42A3/oar-mir-541.pdf]

miRBase precursor : oar-mir-541
Total read count : 584
oar-miR-541-5p read count : 565
oar-miR-541-3p read count : 18
remaining reads : 1

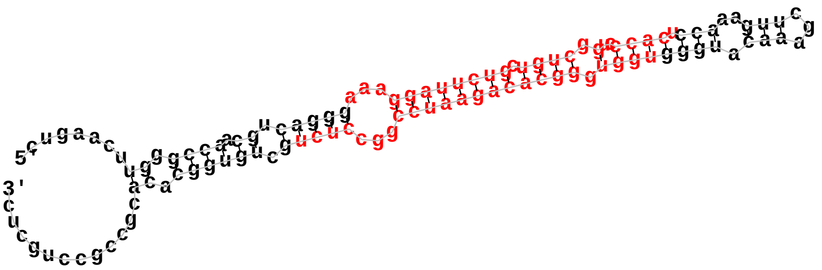

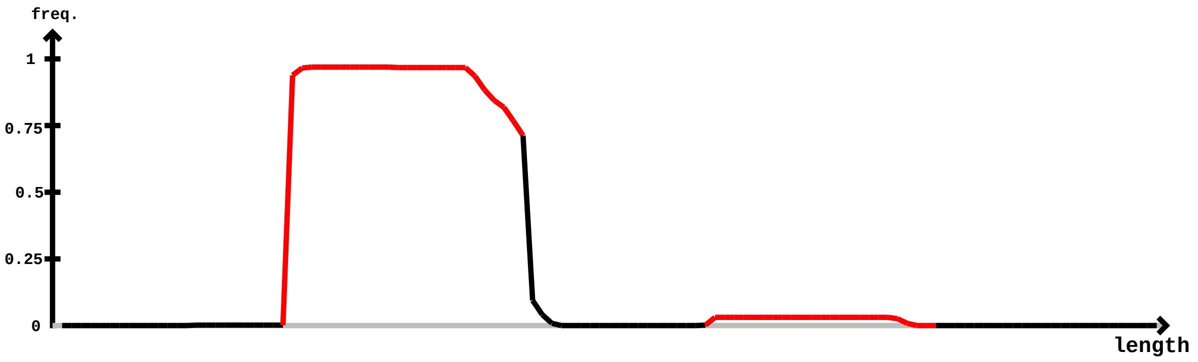


```
                                                                             oar-miR-541-3p
                       oar-miR-541-5p
5'- cugaacuugggccaacgucagggaaaggauucugcugucggucccacuccaaaguucgaaacauggguggugggcacagaauccggccucugcuguggcacacgccgccugcuc   -3'    exp
    ........((((..(((.(((((...((((((((.((((.(..((((.(((..(((...))).)))))))).)))))))))))))...))))).)))(((....)))))))....    reads    mm    sample
    .............aacgucagggaaaggauucug................................................................................    1        0     seq
    .......................aaaggauucugcugucggu........................................................................    19       0     seq
    .......................aaaggauucugcugucgguc.......................................................................    30       0     seq
    .......................aaaggauucugcugucggucc......................................................................    23       0     seq
    .......................aaaggauucugcugucgguccc.....................................................................    15       0     seq
    .......................aaaggauucugcugucgguccca....................................................................    30       0     seq
    .......................aaaggauucugcugucggucccac...................................................................    32       0     seq
    .......................aaaggauucugcugucggucccacu..................................................................    346      0     seq
    .......................aaaggauucugcugucggucccacuc.................................................................    29       0     seq
    .......................aaaggauucugcugucggucccacucc................................................................    19       0     seq
    .......................aaaggauucugcugucggucccacucca...............................................................    4        0     seq
    ........................aaggauucugcugucggucccacu..................................................................    13       0     seq
    ........................aaggauucugcugucggucccacuc.................................................................    2        0     seq
    ........................aaggauucugcugucggucccacucca...............................................................    1        0     seq
    .........................aggauucugcugucggucccacu..................................................................    2        0     seq
    ..................................................................guggugggcacagaauccggcc..........................    1        0     seq
    ...................................................................uggugggcacagaauccgg............................    3        0     seq
    ...................................................................uggugggcacagaauccggc...........................    10       0     seq
    ...................................................................uggugggcacagaauccggcc..........................    4        0     seq
```
